# Evaluation of the 1000 renal transplants carried out at the University Hospital of the Botucatu Medical School (HCFMB) - UNESP and their evolution over the years

**DOI:** 10.1590/2175-8239-JBN-3871

**Published:** 2018-06-04

**Authors:** Hong Si Nga, Luis Gustavo Modelli Andrade, Mariana Moraes Contti, Mariana Farina Valiatti, Maryanne Machado da Silva, Henrique Mochida Takase

**Affiliations:** 1 Universidade Estadual Paulista, Faculdade de Medicina de Botucatu, Departamento de Clínica Médica, Botucatu, SP, Brazil.

**Keywords:** Kidney Transplantation, Immunosuppression, Survival Analysis, Clinical Evolution, Transplante de Rim, Imunossupressão, Análise de Sobrevida, Evolução Clínica

## Abstract

**Introduction:**

The progress in kidney transplantation has been evident over the years, as well as its benefits for patients.

**Objectives:**

To evaluate the 1.000 kidney transplants performed at the Botucatu Medical School University Hospital, subdividing the patients in different periods, according to the current immunosuppression, and evaluating the differences in graft and patient survival.

**Methods:**

Retrospective cohort analysis of the transplants performed between 06/17/87 to 07/31/16, totaling 1,046 transplants, subdivided into four different periods: 1) 1987 to 2000: cyclosporine with azathioprine; 2) 2001 to 2006: cyclosporine with mycophenolate; 3) 2007 to 2014: tacrolimus with antimetabolic; and 4) 2015 to 2016: tacrolimus with antimetabolic, with increased use of the combination of tacrolimus and mTOR inhibitors.

**Results:**

There was an increase in the mean age of recipients and increase in deceased donors and their age in the last two periods. There was a reduction in graft function delay, being 54.3% in the fourth period, compared to 78.8% in the first, *p* = 0.002. We found a reduction in acute rejection, being 6.1% in the last period compared to 36.3% in the first, *p* = 0.001. Urological complications and diabetes after transplantation were more frequent in the first two periods. The rates of cytomegalovirus infection were higher in the last two periods. There was an improvement in graft survival, *p* = 0.003. There was no difference in patient survival, *p* = 0.77 (Figure 2).

**Conclusion:**

There was a significant increase in the number of transplants, with evolution in graft survival, despite the worsening in the profiles of recipients and donors.

## RESUMO

**Introdução:**

O progresso no transplante renal tem sido evidente ao longo dos anos, assim como seus benefícios para os pacientes.

**Objetivos:**

Avaliar os 1000 transplantes renais realizados no Hospital das Clínicas da Faculdade de Medicina de Botucatu, subdividindo os pacientes em diferentes períodos, de acordo com a imunossupressão vigente, e avaliar as diferenças em relação à sobrevida do enxerto e do paciente.

**Métodos:**

Análise da coorte retrospectiva dos transplantes realizados entre 17/06/87 a 31/07/16, totalizando 1046 transplantes, subdivididos em quatro diferentes períodos: 1) 1987 a 2000: ciclosporina com azatioprina; 2) 2001 a 2006: ciclosporina com micofenolato; 3) 2007 a 2014: tacrolimo com antimetabólico; e 4) 2015 a 2016: tacrolimo com antimetabólico, com aumento do uso da combinação de tacrolimo com inibidores da mTOR.

**Resultados:**

Houve aumento da idade média dos receptores e aumento de doadores falecidos e da idade destes nos dois últimos períodos. Observou-se redução de retardo de função do enxerto, sendo de 54,3% no quarto período, em comparação a 78,8% no primeiro, *p* = 0,002. Observamos redução de rejeição aguda, sendo 6,1% no último período em comparação a 36,3% no primeiro, *p* = 0,001. As complicações urológicas e o diabetes após o transplante foram mais frequentes nos primeiros dois períodos. As taxas de infecção por citomegalovírus foram maiores nos dois últimos períodos. Houve melhoria na sobrevida do enxerto, *p* = 0,003. Não houve diferença na sobrevida do paciente, *p* = 0,77 (Figura 2).

**Conclusão:**

Houve aumento significativo no número de transplantes, com evolução na sobrevida do enxerto, apesar da piora no perfil dos receptores e doadores.

## INTRODUCTION

Renal transplantation is the treatment of choice for the majority of patients with chronic kidney disease (CKD),^1^^,^ 2 ^,^ 3 conferring better survival and long-term quality of life when compared to patients undergoing dialysis.^4^^,^ 5 ^,^ 6 ^,^ 7 With these evident benefits, its importance in the world scenario is increasing, with incentives and investments in this field.^8^^,^ 9

The Brazilian organ transplantation program, one of the most important public programs in the world, performed over 5,000 kidney transplants in 2015, ranking Brazil as the second country in absolute number of transplants that year.^10^ Its rise has been progressive since 2006, but with a stalled rate of kidney transplants since 2015, mainly because of the decline in transplants from a deceased donor.^11^ Other figures also deserve attention, such as the number of patients on the waiting list, which was still around 50% of the total transplants performed in the country in 2015.^11^

The HC transplant service at Botucatu Medical School began in 1987, with a progressive increase in the last decade, culminating in 600 transplants in the year 2011,^12^ until reaching the milestone of 1000 transplants in 2016.

The progress of kidney transplantation is due to a number of factors, including the very importance of transplantation as an alternative treatment for patients with CKD, treatment availability as a result of better maintenance of potential donors, family consent^11^ and access to the Unified Health System for all in the country,^11^ in addition to advances in the surgical techniques in the last 50 years, better knowledge about the immunosuppressive therapy used, and the introduction of new immunosuppressive agents.^9^

There were also some changes in the profile of donors and recipients, as well as an adaptation of the services in relation to these changes,^8^^,^ 13 with the management of comorbidities and the aging of this population, and by the contribution of new scientific progresses, including breaking the immunological barrier and viral prophylaxis.^9^

## OBJECTIVE

The goal of the present study was to evaluate the 1,000 kidney transplants performed at the University Hospital of the Botucatu Medical School, State University of São Paulo (UNESP), subdividing the patients in different periods, according to the current immunosuppression, and evaluating the differences in relation to graft and patient survival.

## MATERIALS AND METHODS

An analysis of the retrospective cohort of all renal transplants performed at Botucatu Medical School University Hospital between June 17, 1987, when the first renal transplant of the UNESP HC was performed on 07/31/2016, totaling until then a number of 1,046 transplants. The patients younger than 18 years were taken off the study. The patients were divided according to the predominant immunosuppressive scheme used in the four different periods:

1) 1987 to 2000: combination of cyclosporine with antimetabolic predominant azathioprine. At that time, no induction therapy was used.
2) 2001 to 2006: combination of cyclosporine with mycophenolate predominant antimetabolic. During this period, induction treatment with basiliximab, the drug of choice for the group considered to be of high immunological risk was used: retransplants, children, blacks, and those with a panel > 50%.
3) 2007 to 2014: combination of tacrolimus and antimetabolic. Induction therapy was used in most cases with basiliximab, and with thymoglobulin for patients with a panel > 50%. Induction therapy was not used in transplants with identical or haploidentical living donor.
4) 2015 to 2016: combination of tacrolimus and antimetabolics. Increased use of the combination: tacrolimus with mTOR inhibitors. Use of induction therapy in all cases with basiliximab or thymoglobulin in patients with panel > 30%. Induction therapy was not used only in identical living donors.

### DEMOGRAPHIC DATA

The evaluation was based on the recipients' demographic data, type of dialysis performed before transplantation (hemodialysis, peritoneal or conservative scheme), time on dialysis, baseline disease, donor type (live or deceased). In the case of a deceased donor, the donor's cause of death and age were investigated. The total time of cold ischemia was also considered.

### IMMUNOSUPPRESSION

We also collected data on the immunosuppression used at the time of transplantation, considering the combinations: antimetabolic; cyclosporine with antimetabolic; tacrolimus with antimetabolic and tacrolimus with mTOR inhibitors (imTOR). All schedules were associated with prednisone. Azathioprine or mycophenolate were considered for antimetabolics. For mTOR inhibitors: everolimus or sirolimus.

For induction therapy, we considered basiliximab or thymoglobulin, or nothing at all was considered. The basiliximab dose was 20 mg intravenously on the day of transplantation (D0), during intraoperative and a second dose of 20 mg on D4. For thymoglobulin, we used the total dose of 4.5 mg/kg.

All organs were preserved in Eurocollins solution and no infusion machines were used.

### OUTCOME DATA

The number of acute rejection episodes in the first 6 months, the incidence of cytomegalovirus infections, the number of urological complications and the incidence of diabetes after transplantation were evaluated for each patient.

No protocol biopsies were performed, which indication was guided by the clinical presentation, among the main ones: no graft function in the first 7-10 days after renal transplantation, worsening of renal function without an identifiable factor, proteinuria > 1g, clinical suspicion of viral infection (cytomegalovirus, polyomavirus).

As far as urological complications were concerned, we considered: arterial and venous thromboses, renal artery stenosis, lymphocele, urinary fistula and hydronephrosis.

For cytomegalovirus (CMV), disease was considered in the first two periods due to the absence of a diagnostic method of infection by PCR or antigenemia. In this period, the diagnosis was made by biopsy of the affected organ with an immunohistochemical study after clinical suspicion.

The diagnosis of infection was only possible in the after standardization of the pp65 antigenemia test in the year 2012. A positive antigenemia greater than two cells was considered a CMV infection.

Thus, the diagnosis of CMV disease was performed in the first and second periods by biopsy of the affected organ, showing viral inclusion with confirmation by immunohistochemistry. From the third period (2012), the diagnosis of CMV infection was performed by positive antigenemia, and the diagnosis of disease was maintained by biopsy of the affected organ.

The occurrence of graft function delay was evaluated in deceased donors and considered as the need for hemodialysis in the first week.

Deaths and graft losses were recorded, considering death for the general population (living and deceased donor).

### STATISTICAL ANALYSIS

The Kolmogorov-Smirnov (KS) normality test was performed in order to separate the continuous variables into parametric and non-parametric variables. The analysis of the means of the variables with normal distribution in the four groups under study was made through variance (one-way ANOVA) analysis, assuming equal variances between the groups. For the subanalysis of the groups, the Bonferroni post-test was used. For non-parametric variables, the Kruskal-Wallis variance analysis was used. To compare subgroups we used the Dunn's post-test. To analyze categorical variables, we used the chi-square test. Survival curves were constructed using the Kaplan-Maier method and compared by the log-rank test. For graft survival, death was considered a cause of loss. Cox's multivariate analysis was performed, with graft survival as the outcome. The Forward Stepwise selection method was used. The most significant variables were considered in the univariate model and included in the model: age of the recipient, baseline disease, reactivity panel, type of donor (live or deceased), donor age, cold ischemia time, graft function delay, rejection, cytomegalovirus, urological complications, post-transplant diabetes, induction therapy and baseline immunosuppression.

The results were considered statistically significant when *p* < 0.05. All analyzes were performed using the statistical software SPSS^®^ version 20.

## RESULTS

A total of 1,046 kidney transplants - 388 from a live donor (37%) and 658 (63%) from a deceased donor were analyzed. There was a progressive increase in the number of transplants performed during the periods, and the transplant rate per month, from the first to the fourth period, was: 0.95; 1.4; 6.1 and 10.2 transplants per month, respectively (Table 1).

**Table 1:** Number of kidney transplants (live and deceased donor) per period, total period duration time and transplant rates per month

	1987-2000	2001-2006	2007-2014	2015-2016
Number of transplants	157	104	591	194
Time (months)	165	73	97	19
Transplant rate	0.95	1.4	6.1	10.2

Transplant rate = (number of transplants/time in months).

### BASELINE CHARACTERISTICS AND PROFILES OF THE PERIODS

The results show a predominance of males during all periods (Table 2). There was an increase in the mean age of the recipient in the last two periods compared to previous periods. The mean age in the fourth period was 48 ± 13 years, and in the first period, 36 ± 12 years, *p* = 0.001. An increase in pre-transplantation hemodialysis therapy and a higher rate of diabetics were observed in the last two periods compared to the first. The number of diabetics was 6.4% in the first period and 17.6% in the last period (Table 2). There was a progressive increase in the percentage of transplants with deceased donors, reaching 82.4% in the last period compared to 33.1% in the first period, *p* = 0.001. For deceased donors, we also noticed a reduction in the percentage of donors causing death from TBI in the last two periods, as well as an increase in the average age of the deceased donor compared to the earlier periods. The mean age of the donor increased from 33 ± 12 years, in the first period, to 41 ± 12, in the later one, *p* = 0.001. There was a reduction in the time of cold ischemia in the last two periods, being 23 ± 4 hours in the fourth period, compared to 32 ± 6 hours in the first, *p* = 0.001. (Table 2).

**Table 2:** Baseline characteristics, immunosuppression and outcome from 1,046 kidney transplants, broken down by the periods

		1987-2000 (A) (n = 157)	2001-2006 (B) (n = 104)	2007-2014 (C) (n = 591)	2015-2016 (D) (n = 194)	*p*
Males		61.1%	54.8%	58.7%	57.7%	
Females		38.9%	45.2%	41.3%	42.3%	
Age (years)		36 ± 12	39 ± 12	46 ± 14^(A.B)^	48 ± 13^(A.B)^	0.001^+^
White race (%)		77.7%^(D)^	68.3%	67.2%	62.7%	0,023^#^
Dialysis type	Conservative	1.9%	9.6%	6.3%	3.1%	
	Hemodialysis	71.3%	76.9%	85.8%^(A)^	87.6%^(A)^	0,001^#^
	Peritoneal	26.8%^(C.D)^	13.5%	8.0%	9.3%	
Dialysis time (months)		24 ± 22	29 ± 27	35 ± 32^(A)^	39 ± 37^(A.B)^	0.001^$^
Baseline disease	Hypertension	19.7%	17.3%	21.9%	22.8%	
	Diabetes	6.4%	6.7%	20.7%^(A.B)^	17.6%^(A)^	
	Glomerulonephritis	44.6%^(C.D)^	37.5%^(C.D)^	20.2%	20.2%	0.001^#^
	Undetermined	22.3%	25.0%	24.9%	28.5%	
	Urological	4.5%	6.7%	3.6%	3.1%	
	Others	2.5%	6.7%	8.8%	7.8%	
Class I Reactivity Panel class I (%)		22 ± 14	6 ± 12	11 ± 24	15 ± 29	0.06^#^
Retransplant (%)		3.2%	2.9%	5.8%	4.1%	0. 37^#^
Deceased donor		33.1%	26.0%	70.9%^(A.B)^	82.4%^(A.B.C)^	0.001^#^
Causa mortis	HI	66.7%^(B.CD)^	32.0%	40.4%	40.3%	
	AVE	27.5%	60.0%^(A)^	50.8%^(A)^	45.9%	0.009^#^
	Tumor	0.0%	0.0%	1.8%	0.6%	
	Others	5.9%	8.0%	7.1%	13.2%	
Donor age (years)		33 ± 12	36 ± 11	41 ± 12^(A.B)^	41 ± 12^(A.B)^	0.001^+^
Cold ischemia time (hours)		32 ± 6^(B,C,D)^	28 ± 5^(C.D)^	23 ± 5	23 ± 4	0.001^$^
Induction	Absent	98.7%^(B.C.D)^	60.6%^(C.D)^	17.6%^(D)^	1.6%	
	Basiliximab	1.3%	39.4%^(A)^	64.0%^(A.B.D)^	31.6%^(A)^	0.001^#^
	Thymoglobuline	0.0%	0.0%	18.4%	66.8%^(C)^	
Immunosuppression	antiMET	28.7%^(B.C.D)^	14.4%^(C.D)^	4.6%	1.6%	
	CSA + antiMET	71.3%^(B.C)^	50.0%^(C)^	0.2%	0.0%	0.001^#^
	Tac + antiMET	0.0%	35.6%	92.9%^(B.D)^	63.2%^(B)^	
	Tac + imTOR	0.0%	0.0%	2.4%	35.2%^(C)^	
Delayed graft function		78,8%^(D)^	84%^(D)^	61.6%	54.3%	0.002^#^
Rejection		36.3%^(C.D)^	37.5%^(C.D)^	22.0%^(D)^	6.1%	0.001^#^
Cytomegalovirus		5.7%	14.4%	21.8%^(A)^	42.5%^(A.B.C)^	0.001^#^
Urological complications		26,1%^(C)^	25.0%^(C)^	10.7%	14.8%	0.001^#^
Post-transplant diabetes		13,4%^(C)^	15.4%^(C)^	4.7%	7.1%	0.001^#^
Time of follow up (months)		109,9 ± 98^(C,D)^	99.7 ± 62^(C.D)^	44.2 ± 29^(D)^	8.7 ± 6	0.001^#^
Graft loss		74.5%^(B.C.D)^	45.2%^(C.D)^	26.9%^(D)^	15.5%	0.001^#^
Death		36.9%^(C.D)^	33.7%^(C.D)^	17.5%^(D)^	8.9%	0.009^#^

Legend: antiMET: antimetabolic (azathioprine or mycophenolate); CSA: cyclosporine; TAC: tacrolimus; imTOR: mTOR inhibitors (sirolimus or everolimus). Statistics: $: chi-square;

### IMMUNOSUPPRESSION IN PERIODS

We observed an increase in induction therapy in the last two periods. Basiliximab was predominant in the third period and thymoglobulin in the fourth. In the first and second periods, the absence of induction therapy predominated.

In the first period, the predominant immunosuppression used was cyclosporine associated with antimetabolic and prednisone in 71.3% of the cases, and this immunosuppression was predominant in the second period, in 50.0% of the cases. In the third and fourth periods, the predominant immunosuppression was tacrolimus associated with antimetabolic and prednisone, respectively, in 92.9% and 63.2% (Table 2).

### OUTCOMES IN THE PERIODS

There was a reduction in the percentage of graft function delay in the last two periods compared to the first periods: 54.3% in the fourth period compared to 78.8% in the first period, *p* = 0.002. We also found a reduction in the rates of progressive acute rejection in the periods: 6.1% in the last period compared to 36.3% in the first, *p* = 0.001. Urological complications and diabetes after transplantation were more frequent in the first two periods compared to the later periods. Rates of cytomegalovirus infection were higher in the last two periods compared to the first (Table 2).

### ANALYSIS OF CYTOMEGALOVIRUS INFECTION

Dividing cytomegalovirus infections according to immunosuppression and induction therapy, we found a higher frequency of CMV with induction therapy. As for non-induction, basiliximab and thymoglobulin respectively: 9.3%; 23% and 40.2%, *p* = 0.001 (Table 3). However, the use of induction therapy also depends on the type of concomitant immunosuppression used, which is higher in the tacrolimus with antimetabolic and lower in the tacrolimus group with imTOR (Table 3). In the tacrolimus and antimetabolic regimen, the use of induction therapy was associated with a higher rate of CMV, respectively, for the groups without induction, basiliximab and thymoglobulin, respectively: frequencies of: 10.8%, 24% and 52.1%, respectively, *p* = 0.001. In the tacrolimus with imTOR regimen, the use of induction therapy was not associated with a higher rate of CMV infection (Table 3).

**Table 3:** Incidence of cytomegalovirus (infection and disease) in the four periods by the different immunosuppression regimens and the induction therapy

Immunosuppression regimen	Induction therapy				*p*
	Absent (A)	Basiliximab (B)	Thymo (C)	Total per regimen	
antiMET	6.9%		0.0%	6.7%	0.63^$^
CSA + antiMET	9.7%	9.5%		9.7%	0.97^$^
Tac + antiMET	10.8%	24.0%^(A.C)^	52.1%^(A.B.C)^	29%	0.001^$^
Tac + imTOR		17.4%	5.7%	9.2%	0.10^$^
Total per induction therapy	9.3%	23.0%^(A.C)^	40.2%^(A.B.C)^	22.3%	0.001^$^

Legend: antiMET: antimetabolic (azathioprine or mycophenolate); CSA: cyclosporine; TAC: tacrolimus; imTOR: mTOR inhibitors (sirolimus or everolimus).Statistics: $: chi-square;

### OVERALL SURVIVAL ANALYSIS

**Figure 1:**
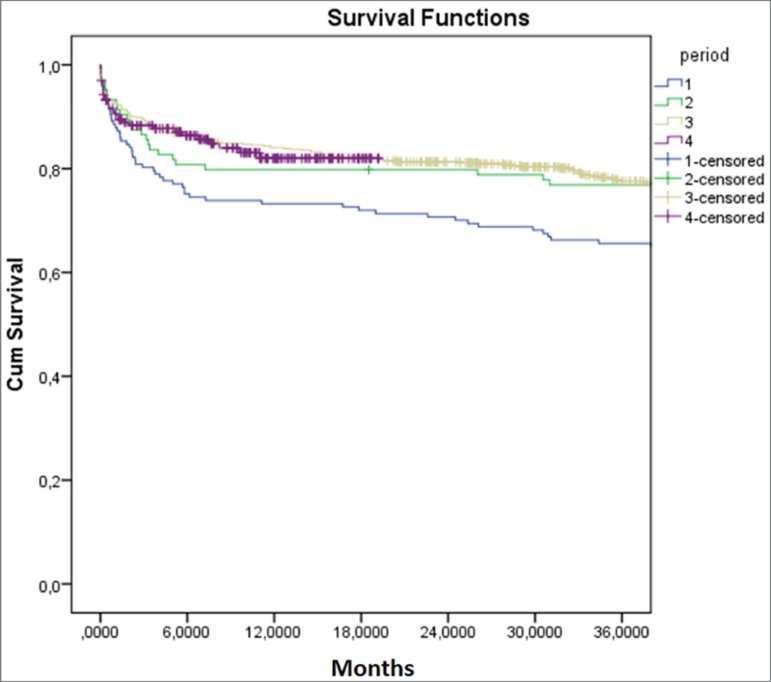
Graft survival in the different periods.

Graft survival at 12, 24 and 36 months were 73.3%, 70.1% and 65.6% in the first period; 79.8%, 78.8% and 75.9% in the second period, 83.9%, 81.3% and 77.6% in the third period, respectively; and 82% in 12 months in the fourth period. There was better survival in the last two periods compared to the first two, *p* = 0.003 (Figure 1).

**Figure 2:**
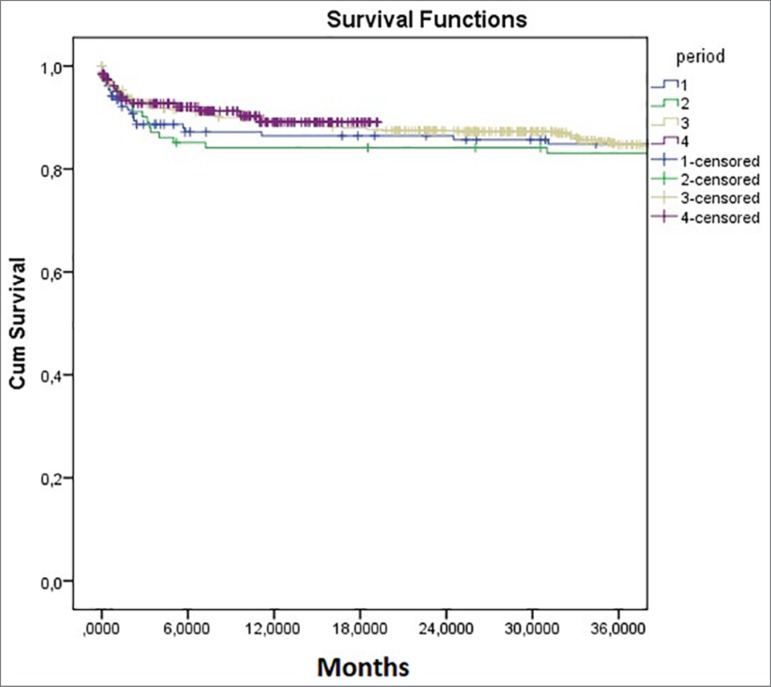
Patient survival in the different periods.

Patient survival at 12, 24 and 36 months were: 86.9%, 85.7% and 84.9% in the first period; 84.1%, 84.1% and 83.1% in the second period, 89.3%, 87.4% and 84.7% in the third period, respectively; and 89.1% in 12 months in the fourth period. There were no differences in patient survival during the periods, *p* = 0.77 (Figure 2).

### MULTIVARIATE ANALYSIS

The Cox's multivariate analysis of risk factors associated with graft loss showed that independent factors were: reactivity panel score, higher donor age, longer cold ischemia time, presence of urological complications, and CMV infection. Protection factors were the use of basiliximab or thymoglobulin induction therapy (Table 4).

**Table 4:** Cox's multivariate analysis of factors associated with a worse graft survival

	OR	95.0% CI for OR		*p*
		Lower	Upper	
Panel	1.012	1.002	1.022	.015
Donor age (years)	1.026	1.011	1.042	.001
Cold ischemia time (hs)	1.068	1.029	1.108	.000
Induction				.006
Induction: Basiliximab	0.429	0.212	0.868	.019
Induction: Thymoglobulin	0.265	0.116	0.609	.002
Cytomegalovirus	1.734	1.089	2.761	.020
Urological complications	2.184	1.322	3.609	.002

Variables included in the model and removed from the equation: recipient's age; donor type; baseline disease; immunosuppression, rejection, graft function delayed post-transplant diabetes.

## DISCUSSION

This study is a continuation of a previous study that evaluated 600 kidney transplants carried out in 2011, and analyzed the differences between three periods, subdivided according to type of immunosuppression. An improvement in survival in the most recent periods was attributed to an improvement in immunosuppressive therapy and team experience.^12^

When completing 1,000 kidney transplants performed, the need arose to subdivide these patients into another period, since in 2015, with the new protocol used in the service, there was an increase in the use of imTOR. An increase in the number of transplants/month was observed progressively, and from 2013, we surpassed the average of more than 100 transplants carried out per year, an amount reached by less than 10% of the 125 centers in Brazil.^11^

This increase in the number of transplants is mainly due to deceased donor transplants, which went from 33.1% in the first period to 82.4% in the last period (2015-2016). Different information was reported by the Brazilian Transplantation Register, which showed constant growth until 2014 (29.6 per million population-pmp), with a slight decrease in 2015 (27.5 pmp), and again in 2016 (26, 8 pmp), which was attributed to transplants with a deceased donor (2.8%); whereas live donor transplants presented little growth (1.7%).

The graft survival at 12, 24 and 36 months was better in the last period, and similar survivals are reported by the Brazilian Transplantation Registry, from 2015,^10^ but slightly higher rates are obtained in the American and European centers, with graft survival rates close to 90%^14^ at 12 months.

In spite of the data presented, that is, a predominance of transplants from deceased donors, with a reduction in the cause of death due to head trauma and a higher average donor age, the results of graft survival are currently better, calling into question the absence of long-term survival. Thus, the results show that elderly patients with a higher rate of diabetes patients are transplanted, with donors with different profiles, and nevertheless obtaining better graft survival.^1^^,^ 8 ^,^ 15

Huang and colleagues evaluated a cohort of 189,944 patients undergoing kidney transplantation in the United States from 2001 to 2013, and compared the glomerular filtration rate (GFR) after one year of transplantation in these different periods. Although the difference in the GFR of these patients was minimal, the changes in receiver's profile, such as age, greater reactivity score and greater prevalence of diabetes mellitus (DM) over the years and expanded criterion organs. In contrast to the worsening of this profile, the changes in immunosuppression presented as an independent factor of better GFR.^16^

The more potent immunosuppression regimen, as well as an increased use of induction therapy, led to a significant decline in acute rejection rates.^17^^,^ 18 In periods 1 and 2, these rates were greater than 35%, dropping to 22% in the third period and 6% in the fourth period - higher than the figures reported in the literature, which varied from around 20% to 10% at present.^16^^,^ 19 It should be considered that the fourth period corresponded to a shorter follow-up period (12 months) compared to other periods, a fact that may have influenced the results obtained.

In the multivariate analysis, the variable associated with a better renal outcome was induction therapy (basiliximab or thymoglobulin use), as previously demonstrated in this study,^20^ and similar results were found by de Castro et al.^21^

The prevalence of cytomegalovirus (CMV), with an exponential progression from 5.7% to 14.4%, in the first periods, and 21.8% and 42.5%, increased by means of immunosuppressive potency and better diagnostic methods in the last periods. This fact can be explained by the lack of a more precise diagnostic method in the first periods in this service. The pp65 antigenemia test was only standardized in 2012; prior to that, the diagnosis of CMV disease was performed by tissue biopsies. However, the increased incidence of CMV is also justified by the more potent immunosuppressive therapy over the years, as well as the more frequent use of thymoglobulin in induction therapy.^22^^,^ 23

In contrast, this high incidence of CMV is not observed when we used imTOR in immunosuppression. Our data show that the use of thymoglobulin as an induction therapy has an incidence of CMV (infection and disease) of 40.2%, but when imTOR is added to the immunosuppressive therapy, this incidence decreases to 5.7% despite the use of thymoglobulin. Previous work has already demonstrated a reduced incidence of CMV with the use of imTOR^24^ because of its antiviral properties,^25^ as in a study by Tedesco-Silva et al.^26^ The incidence of CMV (infection/disease) was lower in the everolimus group than in the mycophenolate group (4.7 *vs*. 10.8 *vs*. 37.6%, *p* < 0.001), corroborating with the data presented, which demonstrated that despite the wide use of thymoglobulin induction therapy, the use of imTOR seems to be a factor of protection for CMV infection, reducing its incidence.^26^

The importance evaluating for CMV is due to the fact that it is one of the main infections in the post-transplantation period, responsible for the morbidity increase,^22^ and its incidence appeared as an independent factor of worse renal outcome in this study.

We also found the panel as an independent factor of worse outcome in renal survival. As already shown in the literature, sensitized patients have a worse outcome in transplantation as non-sensitized patients.^27^ In addition, the donor's age was associated with a worse evolution, as already found in a previous study carried out in this service, which evaluated kidney transplants with donors in acute renal injury. The donor's age was the only characteristic associated with a worse outcome.^28^

## CONCLUSION

Kidney transplant has progressed the world over, and it was from this optimistic panel that the HC transplant service of the Botucatu Medical School was shaped, and efforts have been made to improve the service and increase the number of transplantations, with a primary focus on the outcome and quality of life of the patients.
